# Editorial: Drug-resistance in cancer cells: A new wine in an old bottle

**DOI:** 10.3389/fonc.2023.1161008

**Published:** 2023-02-22

**Authors:** Leonardo Marques da Fonseca, Takeo Tatsuta, Seung-Yeol Park, Leonardo Freire-de-Lima

**Affiliations:** ^1^ Universidade Federal do Rio de Janeiro, Instituto de Biofísica Carlos Chagas Filho, Laboratório de Biologia Celular de Glicoconjugados, Rio de Janeiro, Brazil; ^2^ Division of Cell Recognition, Institute of Molecular Biomembrane and Glycobiology, Tohoku Medical and Pharmaceutical University, Sendai, Japan; ^3^ Department of Life Sciences, POSTECH, Pohang, Gyeongbuk, Republic of Korea

**Keywords:** cancer, ATP binding casette transporter, signaling pathways, chemotherapeutic agents, chemoresistance

Cancer is a major challenge to physicians everywhere, and among its malignancy factors, drug resistance, especially multidrug resistance (MDR) phenotypes stand to be one of the most important challenges. This is why we made a call for papers regarding MDR, its causes, and factors that might affect or are directly related to MDR. At the time of writing this editorial, a total of fourteen articles, six review articles and eight original research articles, have been published in this special issue, with over eighteen thousand views up until now.

Negri et al. report that exosomes from resistant hepatocarcinoma cells can induce resistance in sensitive cells in a process that can be reverted by vitamin D. Ye et al., in their work, propose a novel combination therapy strategy to circumvent drug resistance, using a proteolysis targeting chimera (PROTAC) aimed at BCR-ABL1 mutants, characteristic of chronic myeloid leukemia and tyrosine kinase inhibitors. Narayanan et al. provide us with an evaluation of the anticancer potential of sixteen thiazolyl hydrazone derivates of 1-indanone, remarking that one of them, IHT-6, shows promise in the treatment of p53 mutant colorectal cancers, going as far as providing a possible mechanism of action for the drug. Zeng et al., in their research, show that A011, a sigma-2 receptor ligand shows promising toxicity levels against both parental and resistant MCF-7 cells, which is capable of inhibiting ABCB1 transport activity and downregulating ABCG2 expression. A study involving ovarian cancer patients performed by Oplawski et al. shows a correlation between the levels of antigen CA-125 (associated with drug resistance) and patients that have undergone chemotherapy treatment along with surgery. The work of Indorato et al. evaluates mutations in the Eg5 mitotic kinesin and how they translate into resistance phenotypes, predicting that inhibitors may work or not depending on the binding site for Eg5. Ye et al. provide us with an insightful study of drug-delivery strategies for docetaxel and curcumin *via* liposomes, showing that the co-delivery system is more efficient than the free drugs against MCF-7 tumors in mice. The work of Szymczyk et al. shows us that activation of Akt by either the canonical or alternative pathways is essential for the protective effect against drugs affecting tubulin polymerization in cancer cells.

We also received very insightful review articles that put established knowledge in a new perspective. Xia et al. summarize the regulatory mechanisms of m6A modification in the drug therapy of digestive system malignancies. Li et al. supply a compendium of the understanding of the mechanism of ferroptosis based on the System Xc−/GSH/GPX4 axis in the treatment of drug-resistant solid tumors. Barreto et al. provide an insight into the immunophenotypic characteristics and mechanisms of resistance presented by LSCs and also suggest possible alternatives for the treatment of patients. Zhou et al. judiciously review the more recent works on the emerging role and underlying mechanisms of ncRNAs involved in cancer drug resistance and focus on their clinical applications as biomarkers and therapeutic targets in cancer treatment. Cheng et al. review predictive factors for anti-PD-1/PD-L1 immunotherapy in EC, demonstrating resistance mechanisms to PD-1/PD-L1 blockade. The work of Very and Yazidi-Belkoura summarizes recent evidence that cancer therapeutics affect cellular *O*-GlcNAcylation, and reciprocally, that *O*-GlcNAcylation modulates the response of cancer cells to therapies. It also shows the benefits of targeting *O*-GlcNAcylation as a novel therapeutic strategy for cancer.

This compilation of studies provides a great insight into cancer therapy and drug resistance, covering various subjects from the underlying causes of MDR to ways of circumventing it. Readers will undoubtedly benefit from this material.

## Author contributions

LF, TT, S-YP, and LF-d-L wrote the paper. All the authors read and approved the final version of the manuscript.

